# H_3_ histamine receptor antagonist pitolisant reverses some subchronic disturbances induced by olanzapine in mice

**DOI:** 10.1007/s11011-016-9840-z

**Published:** 2016-05-24

**Authors:** Magdalena Dudek, Kamil Kuder, Marcin Kołaczkowski, Adrian Olczyk, Elżbieta Żmudzka, Aleksandra Rak, Marek Bednarski, Karolina Pytka, Jacek Sapa, Katarzyna Kieć-Kononowicz

**Affiliations:** 1 Department of Pharmacodynamics, Jagiellonian University Medical College, 9 Medyczna Street, 30-688 Krakow, PL Poland; 2 Department of Technology and Biotechnology of Drugs, Faculty of Pharmacy, Jagiellonian University Medical College, Krakow, Poland; 3 Chair of Pharmaceutical Chemistry, Faculty of Pharmacy, Jagiellonian University Medical College, Medyczna 9, 30-688, Krakow, Poland; 4 Institute of Automatic Control, Silesian University of Technology, Gliwice, Poland; 5 Department of Pharmacological Screening, Jagiellonian University Medical College, 9 Medyczna Street, 30-688 Krakow, PL Poland

**Keywords:** Pitolisant, Olanzapine, Sedation, Depression-like symptoms, Locomotor activity, Triglycerides

## Abstract

The use of atypical antipsychotic drugs like olanzapine is associated with side effects such as sedation and depression-like symptoms, especially during the initial period of the use. It is believed that the occurrence of these undesirable effectsis mainly the result of the histamine H_1_receptors blockade by olanzapine. In addition, use of olanzapine increases the level of triglycerides in the blood, which correlates with growing obesity. The aim of this study was to investigate the influence of pitolisant – H_3_ histamine antagonist - on subchronic olanzapine-induced depresion-like symptoms, sedation and hypertriglicerydemia. Forced swim test was conducted to determinate depressive-like effect of olanzapine and antidepressive-like activity during the co-administered pitolisant. The test was performed after the first and fifteenth day of the treatment of the mice. The spontaneous activity of the mice was measured on the fourteenth day of the treatment with a special, innovative RFID-system (Radio-frequency identification system) – TraffiCage (TSE-Systems, Germany). Triglyceride levels were determined on the sixteenth day of the experiment after 15 cycles of drug administration. Daily olanzapine treatment (4 mg/kg b.w., i.p., d.p.d) for 15 days significantly induces sedation (*p* < 0.05) and prolongs immobility time in forced swim tests (FST) in mice (*p* < 0.05); and also elevates the level of triglycerides (*p* < 0.05). Administration of pitolisant (10 mg/kg b.w., i.p.) subsequentto olanzapine normalizes these adverse effects. This study presents a promising alternative for counteracting some behavioral changes and metabolic disturbances which occur in the early period of treatment with antipsychotic drugs.

## Introduction

Olanzapine is one of the most commonly used atypical drugs for schizophrenia (Podogrodzka and Jarema 2010), and one of the strongest antagonists of the H_1_ histamine receptors (H_1_R) (Richelson and Souder 2000). The use of atypical antipsychotic drugs like olanzapine is associated with some side effects, such as sedation and depression-like symptoms, especially in the initial period of the use (Podogrodzka and Jarema 2010). Atypical antipsychotics such as olanzapine may also increase the level of triglycerides in the blood (Albaugh et al. 2011; Misiak et al. 2014), which correlates with the development of obesity during the treatment. This very unfavorable side effect may be related to H_1_R (Reynolds and Kirk 2010) or H_3_ receptor (H_3_R) (Deng et al. 2010). Data from literature confirm the sedative effects of olanzapine in rodents, which increases proportionally to the dose (Weston-Green et al. 2011). It is believed that the occurrence of these undesirable effects may be connected with the blockade of H_1_R (Miller 2004) and cholinergic M_1_ receptor (M_1_R) (Kane and Sharif 2008). Therefore, the search for solutions to the problems of sedation and depression-like symptoms occurring in the initial period of atypical antypsychotics use is very significant. Due to the belief, that simple blockade of histamine action, by H_1_R antagonism may be responsible for these effects, the possible solution might be connected with H_3_R ligands. In our research, we have explored one of the H_3_R antagonists – *Pitolisant* (Wakix ®) - as a potential target to minimize the adverse effects accompanying antypsychotic treatment.

The H_3_ receptor is a pre-synaptic autoreceptor that inhibits synthesis andrelease of the histamine, and simultaneously a heteroreceptor that inhibits other neurotransmitters such as serotonin, noradrenaline and acetylcholine (Deng et al. 2010). It belongs to the G-protein-coupled receptor (GPCR) family, with a complex structure and a large number of isoforms (Lovenberg et al. 1999; Hancock et al. 2003; Bakker 2004). The H_3_R are mainly expressed in the central nervous system (CNS), especially in the areas connected with sleep, cognition and homeostatic regulation (Haas et al. 2008). Blockade of the H_3_R in the central nervous system results in increased synthesis and disinhibition of the histamine release (Brabant et al. 2013), a phenomenon explored further in this study.

Many different ligands of H_3_R have been investigated in recent years, and studies are still in progress (Łazewska and Kieć-Kononowicz 2010; Łażewska and Kieć-Kononowicz 2014). One of the most heavily researched compounds is Pitolisant, which is currently being tested in the third phase of clinical trials (Łażewska and Kieć-Kononowicz 2014).Pitolisant is a selective inverse agonist for the H_3_R,which enhances histaminergic activity in the brain of mice (Ligneau et al. 2007). Its efficacy and safety in the treatment of narcolepsy have been recently documented in a large European multicentre double-blind, randomised, parallel-group controlled trial (Dauvilliers et al. 2013). Pitolisant’s wake-promoting activity was evidenced in excessive diurnal sleepiness of patients with narcolepsy, Parkinson’s disease or Obstructive Sleep Apnea/Hypopnea. The activity is characterized by a mean decrease on the Epworth Sleepiness Scale (ESS) by about five units (Schwartz 2011). Moreover, the procognitive activity of Pitolisant makes it a potential target in diseases with cognitive deficits such as schizophrenia, dementia or ADHD (attention deficit hyperactivity disorder) (Schwartz 2011). Without question, its high therapeutic potential is the reason why we chose this compound for our research.

The aim of this study was to investigate the influence of pitolisant – an H_3_ histamine antagonist - on subchronic olanzapine-induced depression-like symptoms, sedation and hypertriglicerydemia.

## Materials and methods

### Animals

Adult female Albino Swiss mice weighing 20–22 g were used in the study. They were kept in environmentally controlled rooms, in standard cages lit by an artificial light for 12 h each day. Animals had free access to food and water except for the time of the acute experiment. The randomly established experimental groups consisted of 12 mice. The groups were used in the forced swim test (6 mice) and in the locomotor studies (6 mice). Spontaneous activity was performed one day before the forced swim test in asubchronic treated group of animals (6 mice). Triglyceride levels were determined only in the mice subjected to locomotor studies (6 mice). All animal care and experimental procedures were carried out in accordance with European Union and Polish legislation acts concerning animal experimentation, and approved by the Local Ethics Committee at the Jagiellonian University in Cracow.

### Experiment methods

#### Forced swim test (FST)

The forced swim test was conducted according to the method described in the available literature (Porsolt et al. 1977; Pytka et al. 2015a; Pytka et al. 2015b). Mice were forced to swim individually in the glass cylinders (height 25 cm, diameter 10 cm) filled with 10 cm of water at a temperature of 23–25 **°**C. The animals were placed into the cylinders for 6 min, and the total duration of their immobility was recorded after a 2-min initial period. The test was performed after the first and fifteenth day of thetreatment of the mice (the first day after administration – acute treatment, or the sixteenth day after the fifteenth administration – subchronic treatment).

#### Locomotor activity

Locomotor activity was recorded with an Opto M3 multichannel activity monitor (MultiDevice Software v1.3, Columbus Instruments, USA) according to the method described by Pytka et al. (Pytka et al. 2015c; Pytka et al. 2016). After being placed into the cages individually, the mice had their activity evaluated between the 2nd and the 6th minute. The time period chosen corresponded with the time interval considered in the FST. Locomotor activity was evaluated as the distance travelled by the animals while attempting to climb upward. The test was performed after the first and fifteenth day of the treatment of the mice (the first day after administration – acute treatment, or the sixteenth day after the fifteenth administration – subchronic treatment).

#### Spontaneous activity

The spontaneous activity of the mice was measured on the fourteenth day of the treatment with a special, innovative RFID-system (Radio-frequency identification system) – TraffiCage (TSE-Systems, Germany). The test was conducted according to the method described in the literature (Dudek et al. 2015a; Dudek et al. 2015b; Dudek et al. 2016). The animals had the transmitter (RFID) subcutaneously implanted, which tracked the time they spent in different areas of the cage. The data obtained were then grouped in a special computer program. Spontaneous activity was measured starting from the fourteenth day of the treatment at 1:00 pm to the fifteenth day at 9.00 am. The mice were housed in cages in threes, with food and water available ad libitum.

#### Biochemical analysis

Triglyceride levels were determined on the sixteenth day of the experiment after 15 cycles of drug administration. The mice were euthanized by cervical dislocation. The blood was collected after decapitation and then centrifuged at 1200 rtf to obtain the serum. To determine triglyceride levels in the serum, standard enzymatic spectrophotometric tests (Biomaxima S.A. Lublin, Poland) were used. The substrate was decomposed with enzymes appropriate for the relevant product, and converted to a colored compound. The coloration was proportional to the concentration. Absorbance was measured at a wavelength of 500 nm.

### Statistical analysis

The results obtained were analysed using a one-way analysis of variance (ANOVA), followed by a Dunnet post-hoc test, with the significance level set at 0.05 (FST and locomotor activity testsor triglyceride levels) or by a Multiple t-test under the assumption that all rows were sampled from populations with the same scatter (spontaneous activity test). The outcomes were expressed as the means ± standard error of the mean (SEM). Graph Pad Prism 6.0 was used for data analysis.

### Drugs, chemical reagents and other materials

Olanzapine or pitolisant were suspended in 1 % Tween 80. The compounds or vehicle were administered intraperitoneally (i.p.) 30 min prior to the acute experiment. In the pitolisant + olanzapine group, pitolisant was administered 15 min before olanzapine. Subchronic treatment was done at about 9:00 am (0.2 ml Tween to control group, pitolisant - 10 mg/kg b.w. to pitolisant group, olanzapine - 2 mg/kg b.w. to olanzapine group, pitolisant- 10 mg/kg b.w. and olanzapine after 15 min- 2 mg/kg b.w. to pitolisant + olanzapine group) and at about 1:00 pm (olanzapine group and pitolisant + olanzapine group). The volume of the vehicle or the drug solutions was 10 ml/kg.

Pitolisant was tested at dose 10 mg/kg b.w. According the literature (Brabant et al. 2013; Uguen et al. 2013), at this dose, it’s active but lacks the adverse effects. Olanzapine was tested at dose 2 × 2 mg/kg b.w., which, according the literature data (Albaugh et al. 2006; Kim et al. 2014), might induce metabolic disorders in rodents. This dose is also suitable for the observation of decreasing in locomotor activity after olanzapine in rodents (Klingerman et al. 2014). Additionally, due to a much shorter half life of olanzapine in rodents compared to man (van der Zwaal et al. 2008; Liebig et al. 2010), this dose was shown to be appropriate to model pharmacodynamic effects of olanzapine in rodents (Kapur et al. 2003; Liebig et al. 2010). The olanzapine was delivered by Adamed Group (Pieńków, Poland). The Pitolisant was synthesized at the Department of Technology and Biotechnology of Drugs, Faculty of Pharmacy, Jagiellonian University Medical College, Cracow, Poland.

## Result

### Depression-like symptoms

**Fig. 1:**
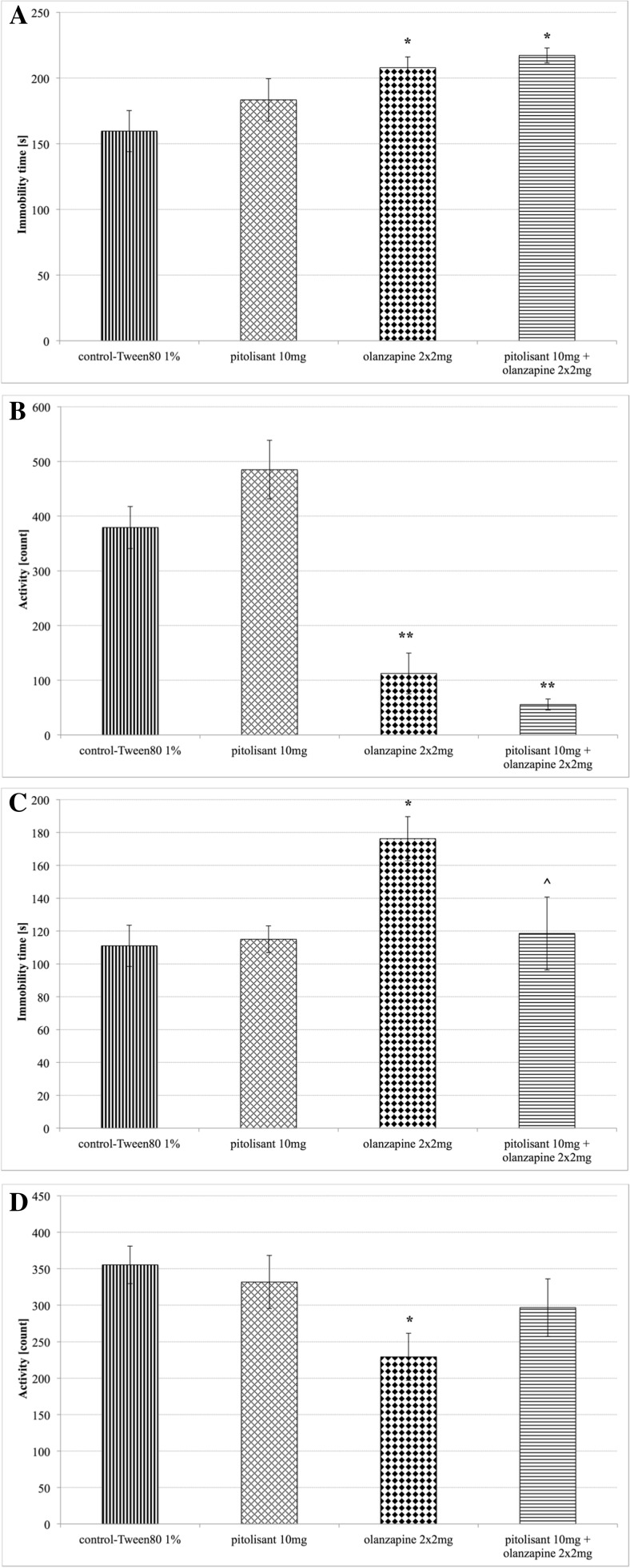
Forced swim test and locomotor activity. **a** Forced swim test after one administration of the compound, **b** Locomotor activity after one administration of the compound, **c** Forced swim test after fifteen administrations of the compound, **d** Locomotor activity after fifteen administrations of the compound, Mean ± SEM, *n* = 6; *P < 0.05 (one-way ANOVA, Dunnet’s post hoc); * - Significant to control group – 0.2 ml 1 % Tween 80, ^ - Significant to olanzapine group – 2 mg/kg b.w. olanzapine in acute tests and 2 × 2 mg/kg b.w. olanzapine in subchronic tests

Pitolisant administered once, and for 15 consecutive days at a dose of 10 mg/kg b.w. did not significantly affect the duration of immobility in the forced swim test in mice. The results are shown in Figs. 1a and c. This compound had also no effect on locomotor activity; neither after one nor after fifteen administrations in mice. The results are presented in Figs. 1b and d.

Olanzapine administered to the mice once at a dose of 2 mg/kg b.w., and for 15 daysat a dose 2 × 2 mg/kg b.w. significantly increased the duration of immobility in the forced swim test in comparison to the control group. The results are shown in Fig. 1a and c. Olanzapine also significantly reduced locomotor activity. The result are shown in Fig. 1b and d.

The administration of pitolisantat a single dose of 10 mg/kg 30 min before a single dose of olanzapine (2 mg/kg b.w.) also significantly affected immobility time in the FST. Subsequent administration of the aforementioned drug sequence in mice statistically significantly increased the duration of immobility in comparison to the time determined in the control group in the FST. It decreased locomotor activity as well. In contrast, the results obtained in subchronic treatment after fifteen administrations of both drugs (pitolisant 10 mg/kg b.w., and after 30 min olanzapine 2 mg/kg b.w., and again after 4 h olanzapine 2 mg/kg b.w.) showed that the administration of pitolisant followed by that of olanzapine equalized the locomotor activity in mice; in comparison to the level of motility in the control group, to which only pitolisant was administered (Fig. 1d). More importantly, this combination of drugs significantly reduced immobility time to the level obtained in the control group in the forced swim test in mice [one-way ANOVA; F_(3,20)_ = 4.226, *P* = 0.0181] (Fig. 1c).

### Spontaneous activity

**Fig. 2:**
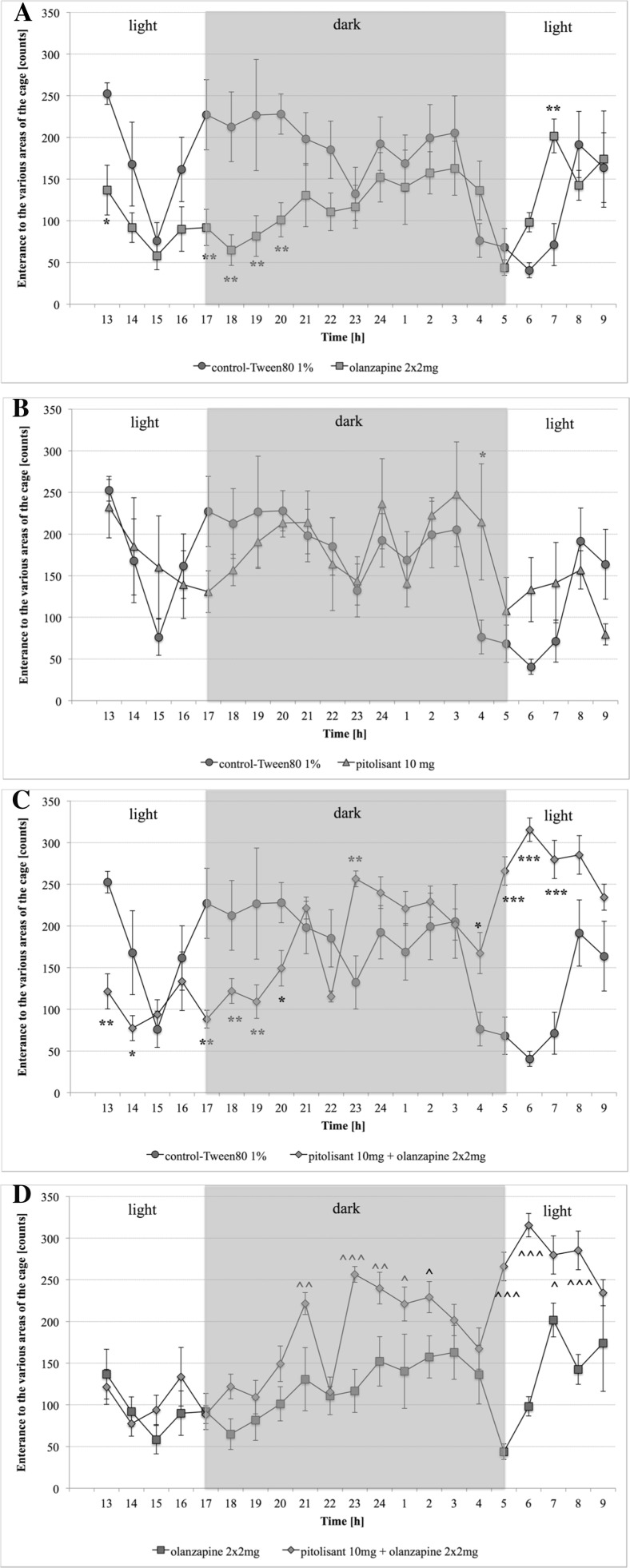
Spontaneous activity after fourteen administrations of the compounds. **a** Comparison of control group to olanzapine group; **b** Comparison of control group to pitolisant group; **c** Comparison of control group to pitolisant + olanzapine group; **d** Comparison of olanzapine group to pitolisant + olanzapine group. Mean ± SEM, *n* = 6; Statistical significant: *P < 0.05 (Multiple t-test)

The control group of mice showed increased motor activity after dark, while during the day their motility was reduced. Both a single administration of olanzapine and its subchronic administration resulted in a statistically significant reduction of spontaneous mobility as compared to the control group in both phases of the day- during the first hour of observation (the bright phase), and from the fifth to the eighth hour of observation (the dark phase). The results are shown in Fig. 2a. Administration of the pitolisant did not significantly affect spontaneous activity in the mice. In the study, the 20-h mobility of the mice treated with pitolisant (10 mg/kg) was very close to the mobility of the mice in the control group, which received onlythe solvent (1 % Tween 80). The results are shown in Fig. 2b. In the group treated with olanzapine and pitolisant together, the mobility was indeed impaired - in the first hours after the second dose of olanzapine, the mice showed reduced motility compared to the control group, but at the end of the dark phase and the following light phase, the mobility was significantly increased. The results are shown in Fig. 2c. When comparing the results of the 20-h observation of spontaneous mobility of the group receiving olanzapine, and the group receiving olanzapine plus pitolisant, it can be said that the addition of pitolisant significantly increases the motility of the mice in the dark phase and the lightphase that follows. The results are shown in Fig. 2d. However, there was significant compensation for the reduced motility of the olanzapine-treated group in the dark phase, i.e. the natural phase of very heavy activity in the mice.

### Triglyceride levels

**Fig. 3:**
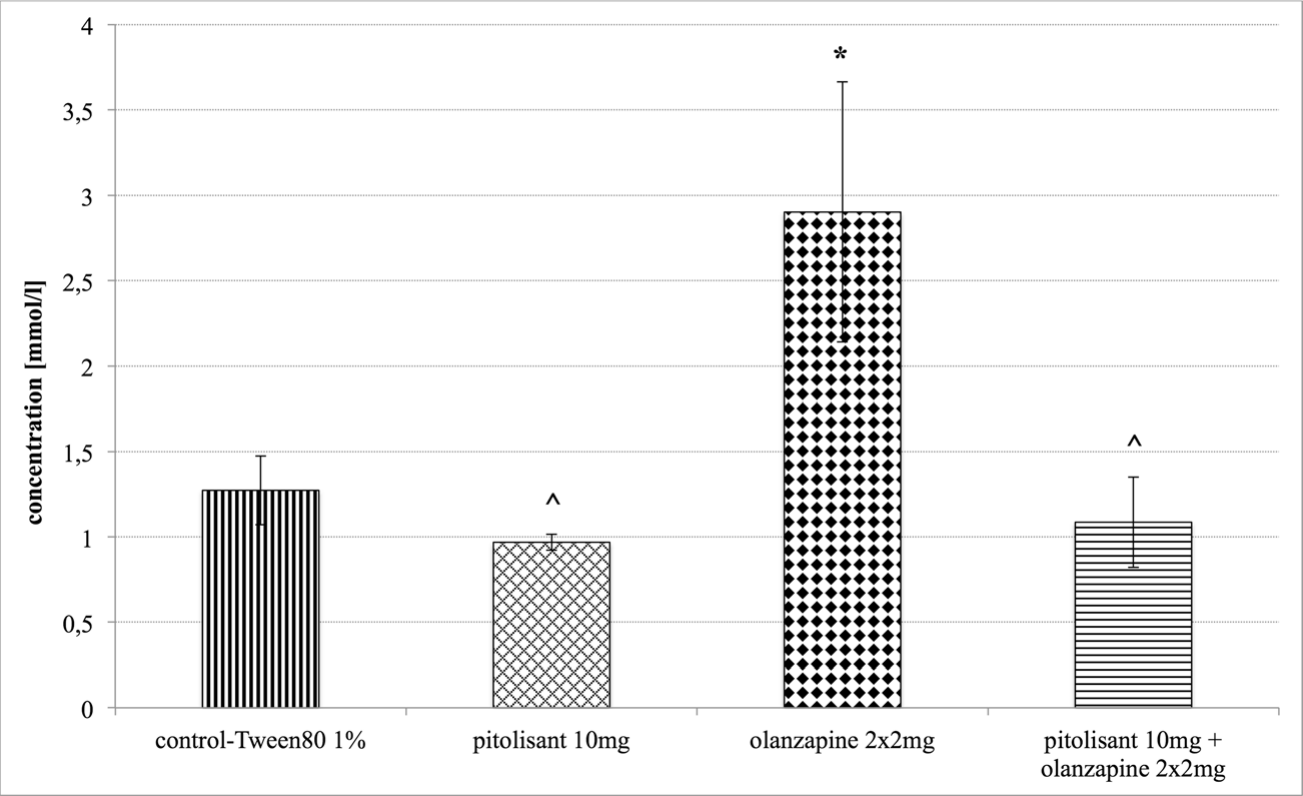
Triglyceride levels. Mean ± SEM, *n* = 6; *P < 0.05 (one-way ANOVA, Dunnet’s post hoc); * - Significant to control group – 0.2 ml 1 % Tween 80, ^ - Significant to olanzapine group – 2 × 2 mg/kg b.w. olanzapine after subchronic treatment

Intraperitoneal administration of olanzapine at a dose 2 × 2 mg/kg b.w. for 14 days resulted in a statistically significant increase in the level of triglycerides in the mice serum. In the group which was administered pitolisant (10 mg/kg b.w.) together with olanzapine, the triglyceride level wascomparable to that in the control mice. Pitolisant administered alone had no effect on the levels of triglycerides in mice. Figure 3 shows the results.

## Discussion

In the current study, the sub-chronic administration of pitolisant in combination with olanzapine resulted in reduction of the immobility time in the FST in mice to the level determined in the control group, which limits depression-like symptoms. Moreover, pitolisant compensated for the spontaneous activity disturbances observed in the olanzapine-treated group. Thus, our results confirm the beneficial effects of the combined used for these compounds.

Studies using various H_3_R ligands have shown that these compounds can favorably affect the levels of triglycerides in animals (Hancock and Brune 2005; Malmlöf et al. 2006). Atypical antipsychotics such as olanzapine may increase the level of triglycerides in the blood (Albaugh et al. 2011; Misiak et al. 2014), which correlates with the development of obesity during the treatment. This very unfavorable side effect may be related to the influence on the H_1_R (Reynolds and Kirk 2010) or H_3_R (Deng et al. 2010). A drug-induced decrease in H_1_R activity may reduce the histamine tone via the H_3_autoreceptors, contributing to the weight gain problem. In addition, atypical antipsychotics may affectfood intake by influencing serotonine, noradrenaline and acetylcholine release via interactions with the H_3_R (Deng et al. 2010). The observed results confirm that the increased response to the histamine by using pitolisant may remove some metabolic disturbances induced by the administration of olanzapine, such as increased triglyceride levels in the serum. This fact is extremely relevant due to the serious adverse metabolic effects which develop in patients treated with atypical neuroleptics.

It is important to mention that there are no reports on the influence of pitolisant on triglyceride levels in literature. Moreover, there is no information available on the combined use of olanzapine and pitolisant in literature to date. Undoubtedly, our preliminary studies with mice show that this is an interesting combination of drugs. Based on the information available, we only know that the co-administration of olanzapine and pitolisant to healthy volunteers does not significantly change the detected plasma level of the drugs in comparison with their individual administration (Schwartz 2011). Additionally, taking into account the results of the Porsolt test and the following motility measurement, we can unequivocally conclude that pitolisant can reduce the olanzapine–induced sedation and depression–like symptoms.

## Conclusion

The observed results confirm that in patients treated with olanzapine who experience unacceptable, adverse effects such as excessive sedation or depression–like symptoms, the administration of pitolisant can be attempted. Moreover, it can be further explored whether this combination of drugs is able to reduce or delay the onset of metabolic disorders induced by olanzapine in patients. The presented preliminary study gives a promising alternative for counteracting some antipsychotic-associated behavioral and metabolic complications, and definitely needs further investigation.
